# Types of Errors Hiding in Google Scholar Data

**DOI:** 10.2196/28354

**Published:** 2022-05-27

**Authors:** Romy Sauvayre

**Affiliations:** 1 Laboratoire de Psychologie Sociale et Cognitive Centre national de la recherche scientifique Université Clermont Auvergne Clermont-Ferrand France; 2 Polytech Clermont Clermont Auvergne INP Aubière France

**Keywords:** reference accuracy, database reliability, false positives, academic publication, research evaluation, scientometrics, citation analysis

## Abstract

Google Scholar (GS) is a free tool that may be used by researchers to analyze citations; find appropriate literature; or evaluate the quality of an author or a contender for tenure, promotion, a faculty position, funding, or research grants. GS has become a major bibliographic and citation database. For assessing the literature, databases, such as PubMed, PsycINFO, Scopus, and Web of Science, can be used in place of GS because they are more reliable. The aim of this study was to examine the accuracy of citation data collected from GS and provide a comprehensive description of the errors and miscounts identified. For this purpose, 281 documents that cited 2 specific works were retrieved via Publish or Perish software (PoP) and were examined. This work studied the false-positive issue inherent in the analysis of neuroimaging data. The results revealed an unprecedented error rate, with 279 of 281 (99.3%) examined references containing at least one error. Nonacademic documents tended to contain more errors than academic publications (U=5117.0; *P*<.001). This viewpoint article, based on a case study examining GS data accuracy, shows that GS data not only fail to be accurate but also potentially expose researchers, who would use these data without verification, to substantial biases in their analyses and results. Further work must be conducted to assess the consequences of using GS data extracted by PoP.

## Introduction

Google Scholar (GS) has become a major bibliographic and citation database. Soon after its creation in 2004, GS received major criticism [1], but subsequently, further studies described it more positively [2,3]. Indeed, the literature acknowledges the free access offered by GS [3-5] and the quality of its coverage [6-12]. The coverage of GS is considered better than that of both Web of Science (WoS) [12-15] and Scopus [9,10], which are GS’s fee-based competitors. This is particularly true regarding its coverage of social sciences and humanities research [10,16,17], conference proceedings [10,14], and books [17]. The GS database has been substantially qualitatively [18] and quantitatively [10,19] improved in all scientific areas such that, according to de Winter et al [18], it could supplant WoS.

However, “the automatic indexing of GS inevitably causes many errors” [20], such as duplicates [21] and false-positive citations [18]. Most researchers generally claim that these errors are negligible [9,10,12,20,22-24], whereas others consider that data cleaning is necessary [16,19,25] but laborious [4,21]. Thus, some scholars have used GS without data cleaning [2,6,11,14,26,27], while others have identified and removed duplicates [4,9,12,17-19,24,28,29]. This removal was performed in 23 of 36 studies (41.8%) using GS data. Furthermore, compared to the authors of related studies, these researchers less frequently identified false positives [17,18,30,31], missing values or omission errors [20,23], document type errors [18,32], author name errors [18,33], publication year errors [16,18,33], title errors [18,33], URL errors [16,32], citation miscounts [32], and inaccessible document errors [30]. None of these 36 studies mentioned any verification of journal names in their data cleaning process. Nevertheless, Haddaway et al [4] attempted to explain the causes of duplicates, showing that they arise from typographical and capitalization errors occurring in journal names. Their findings were confirmed by a study conducted by Valderrama-Zurián et al [34] based on Scopus data.

However, an analysis of 36 articles published between 2008 and 2018 in journals with an impact factor from Journal Citation Reports (JCR) collected from WoS, GS, and relevant studies cited in the most cited research in this field showed that the data verification was not systematically followed by the calculation and reporting of an error rate. Indeed, 14 of the 36 (38.9%) studies explicitly indicated the number or rate of errors. A median error rate of 14.6% with a range from 0.04% to 53.5%, among corpora of citations ranging in size between 127 and 183,596, was calculated. Note that for those studies that were missing error rates but nevertheless had reported adequate results, the error rates were calculated and included. In addition, these studies reported error data of a median of only 1 type of error (range 0-6), and duplicates represented the error type most frequently searched for in this sample of literature (23 of 36).

This median error rate therefore demonstrates that errors are recurrent in GS data. However, GS is a free tool that may be used by researchers to analyze citations; find appropriate literature [35,36]; or evaluate the quality or influence [37] of an author or a contender for tenure, promotion, a faculty position, funding, or research grants [1,21]. Thus, the more an author is cited in a field, the more likely that person is to be considered a highly qualified researcher [38,39]. GS may also be used in research evaluations [23]. Thus, a comprehensive study of this failure of GS may be useful to the scientific community and researchers who want to use this database, whatever their field of study. However, as far as can be seen, no study reports and meticulously quantifies the different types of errors encountered in the GS data extracted by Publish or Perish software (PoP), even though such a study would allow (1) better identification of the limitations of studies based on these data, as described by Hicks et al [40] in the context of research evaluation; (2) enrichment of the thoughtful methodological reflection on potential exposure to GS errors; and (3) development of appropriate methods to limit the negative effects of GS errors on the results produced.

This case study aimed to examine the GS data extracted by PoP, provide a full count of the errors contained in the collected data, and present an epistemological reflection. By doing so, this study offers detailed categorizations of GS data that have not been provided by previous studies. The purpose is especially to address the following questions: (1) What types of GS errors could affect the data and results of researchers’ studies? (2) What methodological problems may result from these errors? (3) How reliable can the citations of GS be without data cleaning?

## Methods

### Context

This GS study is part of broader research that aims to explore the diffusion process of neuroimaging work that sought to alert the scientific community to the issue of false positives. Two references were examined. The first reference is a poster presented at the 15th Annual Meeting for the Organization for Human Brain Mapping (OHBM) [41], and the second is an article published in the short-lived *Journal of Serendipitous and Unexpected Results* (JSUR) [42]. The question was which researchers contributed to this diffusion or, in other words, who cited the OHBM poster or the JSUR article. The collection of citation data became necessary. Nevertheless, some full texts of the citing documents collected by GS did not cite either the OHBM poster or the JSUR article. Thus, this case study was conceived. The reliability of GS data needed to be quantified to identify the limitations of the results produced with GS data before using these data in the diffusion study. This categorization of errors using these 2 references enables one to identify how GS works with literature not referenced by journal editors’ websites. GS uses “automated software, known as *parsers*, to identify bibliographic data” [43] of documents available on the internet. Then, the parser software “typically” collects the same data from full documents without metadata, as the 2 references used in this case study.

### Data Collection

To examine the reliability and accuracy of GS, the citations of both the OHBM poster and JSUR article were analyzed. Note that GS was the only citation database available to collect the citation data for these 2 works because neither WoS nor Scopus indexed them.

PoP version 5 was used to extract references that cited the poster. According to Harzing [44], this software provides a perfect collection of GS data (“Publish or Perish is as accurate or as inaccurate as Google Scholar itself”). In addition, PoP is a common tool in scientometric studies using GS data [14,19,29,45].

The citation data were then collected from GS via PoP. The first author’s name (“Bennett, Craig M”) was entered without quotation marks, and the first part of the OHBM poster and JSUR article title (“Neural correlates of interspecies perspective taking in the post-mortem Atlantic Salmon”) was entered with quotation marks into the “All of the words” software query box. As PoP’s manual explains, the “All of the words” query “matches the search terms anywhere in the searched documents (author, title, source, abstract, references, etc)” [46], as GS does. Thus, this query was used to reproduce the same request with PoP and GS.

This title is so specific that only the following 2 results appeared: (1) 127 references cited the JSUR article [42], and (2) 154 references cited the OHBM poster [41]. In contrast, the reference that appeared in PoP and on GS was a paper supposedly published in a supplement of the famous *NeuroImage* journal and indexed by ScienceDirect. In reality, *NeuroImage* did not publish a journal article written by Bennett et al in 2009 about the neuroimaging work. This *NeuroImage* paper does not exist. What this supplemental issue of *NeuroImage* does contain is the program of the OHBM conference. Therefore, when the citing documents cite the “*NeuroImage* ghost paper,” they actually cite the OHBM poster.

Note that the JSUR article title is almost identical to the OHBM poster title—only the term “proper” in the second part of the title differs. The advantage of this strong similarity is the ability to evaluate the capacity of GS to manage citations of similar references.

A total of 281 references were extracted via PoP on October 6, 2017. Two CSV files were obtained (Multimedia Appendix 1), one for each neuroimaging reference. In this study, several columns that contained the following information were examined: authors, title of the citing document, publication year, publication or source, publisher, and web address of the citing document (“Article URL” as provided by GS). Each column was manually verified, and inaccuracies were counted and categorized in the following 6 steps:

1. The full text of accessible citing documents was downloaded and recorded.
2. The reference list of each citing document was consulted to verify and record the presence of the neuroimaging reference (OHBM poster, JSUR article, or both).
3. The document type was determined and recorded by reading it and searching for additional information on its source.
4. For each citing document, an accurate reference was elaborated for use as a standard and to determine whether GS data contain errors. An inductive and descriptive methodological approach was used to list and identify all the error types that occurred in the GS data. The reference accuracy literature served as a guide to avoid omitting the important errors in this field. A typology was elaborated and presented in the results section as follows: (1) Data collection errors (duplicates, reprints, translations, missing URLs, and inaccessible documents); (2) Academic publication collection errors (retrieval of types of documents other than journal articles, books, book chapters, and conference proceedings); (3) Citation errors (false positives or citation counted by GS when the document does not cite the reference counted); (4) Author errors (missing authors, added authors, missing part of the author’s name, and errors in initials); (5) Title errors (incorrect or incomplete title, and spelling or typographical errors); (6) Publication year errors (erroneous or missing date of publication); (7) Publication of source errors (journal name errors identified in the “Publication” column of GS); and (8) Publisher errors (book editor name errors identified in the “Publisher” column of GS).
5. The GS errors found in each extracted column were listed.
6. The identified errors were aggregated by reference.

This collection, verification, and aggregation process required approximately 170 hours of work.

## Results

### Number of Errors

**Figure 1:**
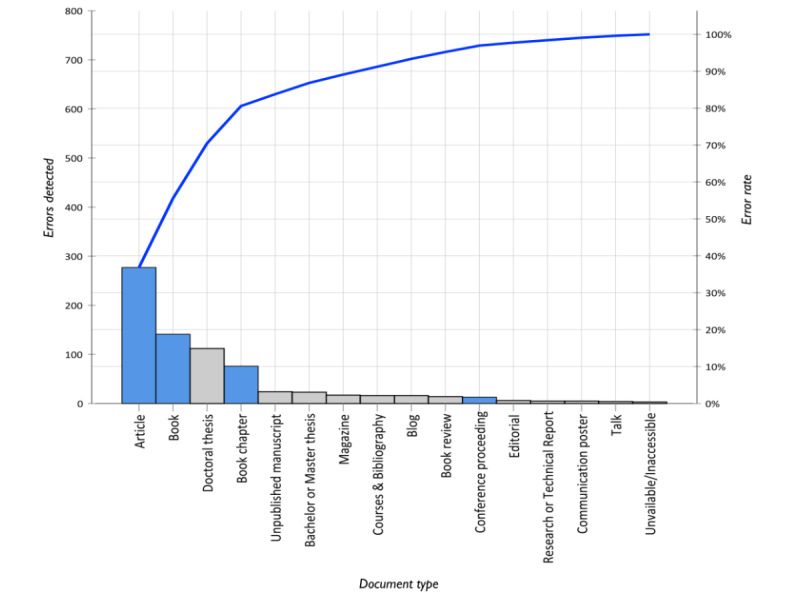
Pareto diagram. Sum of errors detected (N=755) as a function of document type. Light blue indicates academic publications, gray indicates nonacademic documents, and dark blue indicates cumulative sum curve of errors detected.

A total of 755 errors were detected in 281 references retrieved from GS (Figure 1), for an average of 2.7 errors (range 0‑7) (Multimedia Appendix 2). Furthermore, 279 of 281 (99.3%) references contained at least one error.

### Typology of GS Errors

After a manual examination of the references extracted from GS, the following 8 types of errors were identified (Table 1): (1) Data collection errors; (2) Academic publication collection errors; (3) Citation errors (false positives); (4) Author errors; (5) Title errors; (6) Publication year errors; (7) Publication errors; and (8) Publisher errors.

**Table 1:** Typology of Google Scholar errors. Typology and proportion of errors identified as a function of the number of valid references examined and as a function of the total number of errors detected.

Error type	Errors identified, n (%)	
	As a function of the number of valid references examined (n=271-281)	As a function of the total number of errors detected (N=755)
Data collection	42 (5.6)	33 (11.7)
Academic publication collection	77 (10.2)	77 (27.5)
Citation	81 (10.7)	81 (29.9)
Author	61 (8.1)	53 (19.4)
Title	60 (7.9)	57 (20.8)
Publication year	31 (4.1)	31 (11.3)
Publication	155 (20.5)	133 (47.5)
Publisher	248 (32.8)	244 (86.8)

#### Data Collection Errors

Data collection errors included duplicates, reprints, translations, missing URLs, and inaccessible documents (Multimedia Appendix 3). This type of error was identified in 33 of 281 (11.7%) references, and among these errors, duplicates were detected in 16 of 281 (5.7%) references. In addition, URL analysis indicated that none of the GS data in any of the PoP extractions contained duplicate URLs. However, because 18 URLs were missing, a manual search for these references was conducted to obtain and verify them. Among these missing URL references, only 2 of the 18 citing documents were inaccessible, and 9 references were duplicates, translations, or reprints.

#### Academic Publication Collection Errors

Some scientometric studies have used document type as a variable. Consequently, some researchers have focused exclusively on journal articles [3,6,29,30,47,48], whereas others have presented their collected citations per document type, including journal articles, books, book chapters, and conference proceedings [25]. Furthermore, “grey literature” [4], such as theses and research reports [18], can also be included. Considering the diversity of this research method, it will be interesting to further explore the document types that GS is likely to retrieve and count.

GS describes itself as a database that “provides a simple way to broadly search for scholarly literature” [49]. However, what does “scholarly literature” mean for GS? The definition provided by GS, and used in the document inclusion process, encompasses “journal papers, conference papers, technical reports or their drafts, dissertations, preprints, postprints, or abstracts” [43]. On another webpage, GS mentions that users “can search across many disciplines and sources: articles, theses, books, abstracts, and court opinions” [49]. By contrast, GS excludes “news or magazine articles, book reviews, and editorials” [43] because they are “not appropriate” [43]. Nevertheless, there is no statement about the rejection of these undesirable documents from the GS index.

In this study, the document type of each reference collected from GS was examined to determine whether the document in question was an “academic publication.” In this way, a document was considered an “academic publication” only if it was (1) an article that was published in a journal with an International Standard Serial Number (ISSN) or (2) a book, a book chapter, or conference proceedings published with an International Standard Book Number (ISBN). All other document types (thesis, magazine, communication poster, bibliography, course, report, and unpublished document), so-called “nonacademic documents,” were classified as GS collection errors. Note that, according to this definition, a doctoral thesis is an academic work but not an academic publication.

As Multimedia Appendix 4 shows, GS retrieved 203 of 281 (72.5%) academic publications, but included 77 nonacademic documents in the corpus. The error rate reached 27.5% according to the definition given in the literature, whereas the GS definition led to a lower error rate (6.8%). In addition, because GS data are asymmetrically distributed, a nonparametric (Mann-Whitney) test was conducted with SPSS 25 (IBM Corp), and it revealed that the nonacademic documents tended to contain more errors than the academic publications (*U*=5117.0; *P*<.001) (Multimedia Appendix 5).

#### Citation Errors (False Positives)

The reference list of each citing document was examined to determine which of the 2 references (OHBM poster or JSUR article) had been cited. A total of 271 full documents were available and were read. This assessment revealed that 81 of the documents (29.9%) did not cite the reference retrieved from GS. In other words, 29.9% of the citations counted by GS were false positives. In 8 of the 271 (3.0%) cases, neither of the 2 references were found. In 12 of the 271 (4.4%) cases, the JSUR article reference was found instead of the OHBM poster reference, that is, in the extraction of citations attributed by GS to the OHBM poster. Conversely, in 61 of the 271 (22.5%) cases, the OHBM poster reference was found instead of the JSUR article reference.

Additionally, these citation errors (false positives) affected 8 times more OHBM poster references than JSUR article references (odds ratio 7.77, 4.4 < CI < 13.71). Note that the OHBM poster reference was misreferenced in the citing documents more often than the JSUR article reference.

#### Author Errors

As Multimedia Appendix 6 shows, 53 of 273 (19.4%) references contained at least one author error. For example, initials were removed or added. Authors were missing in 41 of the 273 (15.0%) references. Surprisingly, they were replaced by a journal name or by the title of either their own book or their own book chapter. Finally, 104 authors were missing, while 20 authors were improperly added. In summary, 124 of 565 authors (22.0%) were inaccurate.

#### Title Errors

A thorough examination of the “Title” column extracted from GS showed that 57 of 274 (20.8%) references contained at least one error (Multimedia Appendix 7). The incompleteness of the title was the most common error identified. As a result of this error, some incomplete titles were similar to other publication titles. Furthermore, several errors were more questionable, such as replacement of a book title with a chapter title from the aforementioned book or with the title of a different chapter from another book by an author who contributed a chapter to this book. Other questionable title errors were the assemblage of 2-chapter parts published in the same book and the replacement of the publication title by its editor’s name or the domain name of the website that hosts it. Surprisingly, irrelevant parts were added to the publication title, such as an ISBN number, the price of the book, the name of the book collection, and an excerpt from the front page of a thesis (“a dissertation submitted for the degree of Doctor of Philosophy”). Lastly, the reference titles also contained typographical or spelling errors.

#### Publication Year Errors

Publication year errors were detected in 31 of 274 (11.3%) references. In most cases, the years were missing (they were replaced by “zero” in 22 references). In other cases, the actual publication year of the JSUR article or the OHBM poster was increased by 1 year or decreased by 1, 3, 7, or 100 years (Multimedia Appendix 8).

#### Publication of Source Errors

The “Publication” or “Source” column retrieved from GS via PoP showed inconsistencies that depended on the document type of references (Multimedia Appendix 9). Indeed, it contained journal names, books, edited book titles, conference proceeding titles, magazine names, publisher names, domain names of websites that host the citing documents, irrelevant parts of references, and even an author’s address. Furthermore, a large number of missing values (ie, “not provided” in Multimedia Appendix 9) were found in these publication data, affecting 1 in 3 (32.0%) references. These missing publication data were observed most often for theses (bachelor’s, master’s, and doctoral theses) and book references.

In total, 133 of 280 (47.5%) references contained errors Table 2). These errors were mostly identified in conference proceedings, edited books, and journal articles. In addition, only half of the citations counted by GS were usable as academic publication material (Table 2, “Utility” column) because, for example, GS provides a domain name instead of the academic journal name. Among these usable data, 133 were inaccurate. Finally, in this corpus, only 60 of 280 (21.4%) references had proper usable data.

**Table 2:** Accurate and inaccurate content identified in the “Publication” column retrieved from Google Scholar via Publish or Perish (N=280).

Type of error	Inaccurate publication, n (%)	Accurate publication, n (%)	Utility^a^
Journal name (n=108)	56 (51.9)	52 (48.1)	(+)
Magazine name (n=2)	1 (50.0)	1 (50.0)	(−)
Book title (n=13)	13 (100.0)	0 (0.0)	(−)
Edited book title (n=29)	21 (72.4)	8 (27.6)	(+)
Conference proceeding title (n=5)	5 (100.0)	0 (0.0)	(+)
Thesis title (n=2)	2 (100.0)	0 (0.0)	(−)
Publisher name (n=2)	2 (100.0)	0 (0.0)	(−)
Domain name (n=20)	18 (90.0)	2 (10.0)	(−)
Preprint database name (n=4)	1 (25.0)	3 (75.0)	(−)
Other (n=5)	5 (100.0)	0 (0.0)	(−)
Missing value (not provided) (n=90)	9 (100.0)	81 (90.0)	(−)
Total (n=280)	133 (47.5)	147 (52.5)	N/A^b^

^a^The usable publication content for studies using academic publications is denoted by “+.” The errors were not easy to categorize because of nonacademic documents. For instance, when the document type is a blog post or an unpublished draft, a journal name is not expected in the “Publication” column and thus is counted as an inaccuracy. Nevertheless, this type of document had already been counted as a data collection error. Therefore, each document type was specifically analyzed to avoid falsely increasing the error count. However, the categorization was easier for other references, such as when the journal editor name was provided instead of the journal name. In addition, an examination of spelling and typographical errors, including capitalization errors, was conducted.

^b^N/A: not applicable.

These source inconsistencies mainly occurred in journal names as typographical errors, particularly capitalization errors (Multimedia Appendix 10). The second most frequent error was title and journal name incompleteness. Journal names were heavily truncated, as shown in the following examples: “Journal of …” instead of “Journal of Advertising Research” and “Rev …” instead of “Revista de neurologia.” The same type of inaccuracy was identified in the edited book titles as follows: “… Routledge Handbook of …” instead of “The Routledge Handbook of Neuroethics” and “… Imaging of the …” instead of “Imaging of the Pelvis, Musculoskeletal System, and Special Applications to CAD.” Furthermore, as several journal names begin with “Journal of” and several edited books begin with “Routledge Handbook of,” the incompleteness of GS data may cause difficulties.

#### Publisher Errors

The “Publisher” column retrieved from GS provided a variety of content (Multimedia Appendix 11) as follows: editor name (including journal editor), journal name, domain name of the website that hosts the citing document (eg, 42 of the domain names were “books.google.com”), digital library (ie, JSTOR), and missing values. The “Publisher” column contained the highest error rate found in the GS data, which was 244 of 281 (86.8%) references (Table 3). Indeed, the 248 inaccuracies detected in this column constituted a third (32.9%) of the total errors identified. Journal editors and domain names were frequently inaccurate. The utility of this publisher data was then limited to studies using academic publication data. Only the editor names of books, book chapters, and conference proceedings were usable, but they actually represented 35 of the 281 (12.5%) references. Furthermore, an error rate of 37.1% was found in these usable data. For example, an editor’s name was replaced by an irrelevant name (The Penguin Press by Australia Books and Palgrave Macmillan by Springer).

**Table 3:** Accurate and inaccurate content in the “Publisher” column retrieved from Google Scholar via Publish or Perish (N=281).

Type of error	Inaccurate publication, n (%)	Accurate publication, n (%)	Utility^a^
Book and conference proceeding editor (n=35)	13 (37.1)	22 (62.9)	(+)
Journal editor (n=51)	51 (100.0)	0 (0.0)	(−)
Journal name (n=1)	1 (100.0)	0 (0.0)	(−)
Digital library name (n=2)	2 (100.0)	0 (0.0)	(−)
Domain name (n=167)	167 (100.0)	0 (0.0)	(−)
Not provided (n=25)	10 (40.0)	15 (60.0)	(−)
Total (n=281)	244 (86.8)	37 (13.2)	N/A^b^

^a^The usable publication content for studies using academic publication data is denoted by “+.”

^b^N/A: not applicable.

## Discussion

### Principal Findings

The aim of this study was to examine the accuracy of citation data collected from GS via PoP and to provide a comprehensive description of the errors and miscounts identified. In fact, the extraction of raw data with inaccuracies from GS may generate incorrect results in several research areas, such as bibliometrics, scientometrics, and research evaluation. Despite the data cleaning performed by researchers (mainly duplicate removal), citation counts retrieved from GS were generally used without substantial caution. Furthermore, few comprehensive studies listed the different types of GS errors, and no previous research seemed to quantify the inherent problems of GS citations collected by PoP. This study was therefore conducted to provide a meticulous analysis of GS data to anticipate the risk of errors that may affect the data and the results of studies using them.

### Ranking of GS Errors

The GS errors were analyzed using 281 documents that cited a neuroimaging work performed to raise awareness of false-positive results in the scientific community. This study revealed an unprecedented error rate, with 279 of 281 (99.3%) examined references containing at least one error. Academic publications were not free from errors. They accounted for 503 of the 755 (67.0%) detected errors. However, nonacademic documents tended to contain more errors than academic publications (*U*=5117.0; *P*<.001).

The cumulative error rate detected in this study (99.3% of references containing at least one error) differs from the median rate (14.6%) reported in the literature over the past 10 years. This difference may be explained by several aspects of previous research. First, an automatic approach was generally used to clean the data, while a manual examination was conducted in this study. Second, a varied but low number of variables were examined in these studies. A median of 1 type of error was examined in previous studies, while 8 types of errors were examined in this study. Third, the usual purpose of these studies was to compare the coverage of GS, WoS, and Scopus; thus, the researchers mainly verified duplicates in an aggregated corpus drawn from these 3 databases. Fourth, these studies did not cumulate the number of errors identified per reference.

These discrepancies make comparison difficult, but data provided by de Winter et al [18] (“Online Supplementary Material 5 Excel File”) make it possible. Through these data, an error rate cumulated by reference was calculated to compare what is comparable. However, as these researchers used 4 error types, the comparison was performed for academic publication collection errors, author errors, title errors, and duplicates. All other things being equal, this study reports an error rate 3 times higher than that reported by de Winter et al [18] (64.8% and 20.5%, respectively). These findings suggest that citation counts and references extracted from GS are not fully reliable and may expose the researchers who use them to numerous errors. Note that the content of GS is the result of automatic indexing of websites by robots. The coverage depends on the indexed websites. Moreover, according to GS, “robots generally try to index every paper from every website they visit, including most major sources and also many lesser known ones” [50]. Thus, the reliability of GS is a type of “photography” of the reliability of authors’ and editors’ websites. Since errors can happen, it is important to identify the possible impact of GS’s lack of reliability with respect to research data.

### The Impact of GS Errors in Research Data

What is the probable impact of GS errors in the citation analysis or research evaluation area when citation counts and references are used without data cleaning?

#### Publisher Errors

The useful content that a researcher needs to find in the “Publisher” column extracted from GS via PoP is the editor name for books, book chapters, and conference proceedings. However, this column mainly contains the domain name of the website hosting the citing document. Thus, only 7.8% of these collected data are free from errors and are usable in an academic publication study. The “Publication” column therefore requires meticulous examination before use. The first step is to determine the document type of each collected reference because GS still does not provide it.

#### Publication Errors

The PoP manual indicates that the “Publication” column contains “journal name or similar,” and “similar” is not explicitly defined, which is “not always available” and “sometimes wrong” [51]. However, the “publication” content is more disparate and incorrect than this. Indeed, it contains journal names, book titles, thesis titles, publisher names, and domain names, and one-third of this column involves missing values. Only 21.4% of “publication” data are free from errors and are usable in an academic publication study. In addition, GS errors can impact studies in the following ways. First, the GS error rate can negatively affect the evaluation of journal impact factors and the journal ranking. Second, missing values (32.4% of references) can alter relational database management [52]. Third, typographical errors (including capitalization errors) can lead to duplicates [4]. Lastly, note that there is a risk in using the “publication” data because of the large number of errors detected in the journal names, edited book titles, and conference proceeding titles.

#### Citation Errors (False Positives)

The GS citation count is distorted by documents that do not cite the reference retrieved. This point is often reported in the literature, for instance, as a “phantom citation” or “false citation” [20], but no reported error rate [18,30,32,53,54] is as high as the rate found in this study (29.9%). This difference can be explained by the highly similar titles of the 2 references examined (OHBM poster and JSUR article). This finding also demonstrates the difficulties of addressing this type of similarity in GS data. Consequently, researchers may use data samples that contain false-positive citations and then may obtain biased findings.

#### Academic Publication Collection Errors

GS failed to retrieve only academic publications. Indeed, 27.5% of the citing documents were nonacademic publications, including doctoral theses, magazine articles, preprints, reports, courses, bibliographies, and blog posts. This error rate confirms previous findings [30]. However, if the GS definition of “scholarly literature” is applied, this error rate falls to 6.8%. This GS definition differs widely from the definition of “academic publication” used in this study. Thus, GS seems to inaccurately report citation counts and references of academic publications, and consequently, it does not accurately reflect the dissemination of published work. Therefore, the results of many scientometric studies using GS data to examine the publication activity of scientists, particularly in research evaluation, may be questionable when these data are not verified and cleaned (document types and false positives). The citation counts and h-index scores calculated by GS are also questionable. This raises questions about the reliability of studies that compare the coverage of GS, WoS, and Scopus, and conclude that GS collects significantly more citations [12,28,29] than its competitors. Further research should explore the citation counts of these databases to determine how comparable they are.

#### Title Errors

The main issue with the titles retrieved from GS is incompleteness, which causes problems such as false-positive matches. The similarity between the OHBM poster title and the JSUR article title demonstrates this GS difficulty. Other errors (typographical and spelling) cause problems in database management. More unwelcome is the missing title error. Instead of the title, 6.2% of references contained, for example, editor names, domain names, or ISBN numbers. These missing title errors raise several problems as follows: (1) references cannot be retrieved with a search by title, and (2) duplicates can be more frequent and more difficult to detect.

#### Author Errors

The citing documents examined were cowritten by 565 authors. Nevertheless, 124 (22.0%) authors were either incorrect or missing. These errors can cause problems in studies of the structure of scientific collaborative networks, which are commonly used graphs. Indeed, a fifth of the collaborative networks built may be incorrect and thus may generate imperfect relationships. First, the missing authors may truncate an important share of all the authors involved. Second, the irrelevant added authors may create a bias that a graph’s algorithms can reinforce. Consequently, researchers may overestimate a relationship or ignore another determinant one.

#### Data Collection Errors

Duplicates, translations, and reprints are frequent in GS data. As collected data can be biased by duplicates, their detection is the first step implemented in studies using GS data. The duplicate, translation, and reprint rates found in this study were similar to those in previous studies [30,32]. In addition, the URL address of citing documents is commonly used to detect duplicates and collect full-text documents. Because a missing URL may cause difficulties, previous studies resolved this issue by automatically deleting a reference without a web address [16,32]. By contrast, in this study, 6.4% of URL addresses were missing, but only 0.7% of them could not be found with a manual search. Half of these found documents were usable, and half were duplicate, translation, and reprint references. Consequently, the duplicate search removed 7.8% of the references, whereas the irrelevant deletion of references without a URL address led to the omission of 3.2% of the citing documents. Again, this may cause biased results.

#### Publication Year Errors

Incorrect years had a lower frequency than missing years (3.3% and 8.0% of references, respectively). These missing values can cause major problems in data collection. As GS limits the search results to the first 1000 citing references per query, certain researchers have collected data by publication year to obtain a larger corpus of GS citations [25] or to focus their analysis on a specific period of time [45]. Other researchers have removed references containing incorrect publication years [16]. Thus, these neglected references may lead to truncated data and biased results. Inherent to the failed indexation process of GS, this publication year error may cause sampling errors that affect the representativeness of findings.

### Data Verification Versus Biased Results

The GS error rate seems to be negligible when types of errors are considered in isolation. These types of claims have been made about false positives [20], duplicates [10], and incorrect publication years [2]. By contrast, with regard to GS errors, Harzing [55] argues fatalistically that “bibliometrics is an inexact science and that any data source has its own flaws.” However, Hicks et al [40], in presenting the Leiden Manifesto, emphasized the importance of the quality of the data used in research evaluation. Conversely, when the GS error rates are observed as a whole, a worrying cumulative effect is revealed. Indeed, only 2 of 281 (0.71%) references collected from GS were free from errors. This raises a question about the reliability of GS citation counts. In this study, 2 neuroimaging works were cited 281 times according to GS. However, this citation count is incorrect. In fact, these works were cited 131 times in academic publications (ie, excluding duplicates, reprints, translations, inaccessible documents, and false positives), which is 53.4% less than the GS claim. Thus, the full sample collected from GS (281 citations) can considerably differ from the proper sample (131 citations). There is thus a major risk of producing incorrect and biased results that do not accurately reflect the data examined.

Consequently, meticulous verification and cleaning of GS data are essential before using them. Considering this, several precautions should be taken to improve the reliability of GS data. First, detect and remove duplicate, translation, and reprint references and subsequently merge their citation counts. Second, consult the full-text documents of the full sample to remove false-positive matches. Third, verify the document type of each reference to exclude nonacademic publications.

Because results will be biased or wrong if these verification steps are not performed, is it possible to study a large-scale sample of GS citations (approximately several thousand)? It seems unlikely unless substantial resources are allocated for such verification. Indeed, Meho and Yang [21] were allowed 18 minutes per reference (3000 hours of work for 10,000 citation samples). In this study, a work time of 32 minutes per reference was necessary to complete the verification (150 hours for 281 citations). What about automatization of the verification? Studies that cleaned large-scale data either in part or as a whole using an automatic cleaning process [4,10,20] reported a lower error rate and fewer error types than studies using a manual cleaning process [30,54]. Therefore, it is reasonable to have doubts about the efficiency of this automatic cleaning.

Finally, studying a small sample of GS data seems more adequate than studying a large sample in terms of obtaining reliable data and accurate findings. Nevertheless, there is a need to conduct further research to develop statistical tools for weighting the correlation calculation in a large-scale sample of GS data, which are widely used in database coverage studies. However, these tools may not correct the collection issue inherent to the GS database.

Alternatively, according to the reference accuracy literature [9,10,12-15], databases, such as WoS and Scopus, can be used in place of GS because they are more reliable, though they have narrower coverage than GS. Indeed, WoS has an average error rate of 0.1% [4,18,31,32], and this rate is 1.0% for Scopus [10,31]. However, since GS is a free database [56], it may be the only possible way to conduct a study. However, knowing that all databases are likely to contain errors, verifying a sample of data is a useful precaution.

To conclude, the categorization of the errors encountered in the data extracted from GS provides researchers with methodological and epistemological reflections so that they become aware, with precision, of the probable errors that they are likely to encounter, and can consequently adjust their methodological choice. For example, the number of citations obtained by GS may not be completely accurate, or the names of the authors mentioned may not be completely correct. With a sample of several thousand references, these errors can have a noticeable impact on the results.

### Conclusion

Almost all of the data retrieved from GS contained at least one error, calling the reliability of GS data into question. Further, the reliability of studies using a large-scale sample without verification and data cleaning is also called into question. Moreover, studies using GS to evaluate research activity or compare the coverage of several databases (ie, GS, WoS, and Scopus) may be affected by substantial biases, including citation miscounts.

However, researchers who are able to spend a considerable amount of time on the meticulous verification of their small samples can obtain various references for journal articles, books, edited book chapters, and conference proceedings from GS. This ability can be especially useful in bibliometric studies based on material published in research areas in which journal articles are less predominant than other publication types.

### Limitations

Since the data used are limited and specific, the results obtained cannot be generalized. However, this case study provides a kind of “stress test” of GS to promote reflection on the limits of this free database.

## Appendix

Multimedia Appendix 1 Raw data.

Multimedia Appendix 2 Number of errors detected per reference retrieved from Google Scholar.

Multimedia Appendix 3 Data collection error: types and rates.

Multimedia Appendix 4 Document types of references collected from Google Scholar as a function of 3 categories (academic journal, nonacademic journal, and Google Scholar non-“scholarly literature”).

Multimedia Appendix 5 Results of the Mann-Whitney test on the type of reference (academic publication or nonacademic document) and the number of Google Scholar errors, and data visualization with a box plot.

Multimedia Appendix 6 Inaccurate content identified in the “Author” column retrieved from Google Scholar via Publish or Perish software.

Multimedia Appendix 7 Inaccurate content identified in the “Title” column retrieved from Google Scholar via Publish or Perish software.

Multimedia Appendix 8 Inaccurate content identified in the “Year” column retrieved from Google Scholar via Publish or Perish software.

Multimedia Appendix 9 Content of the “Publication” column retrieved from Google Scholar via Publish or Perish software as a function of reference document type.

Multimedia Appendix 10 Spelling and orthographical errors in the academic publications retrieved from Google Scholar via Publish or Perish software: journal name, edited book title, and conference proceedings book title.

Multimedia Appendix 11 Content of the “Publisher” column retrieved from Google Scholar via Publish or Perish software as a function of reference document type.

## Abbreviations

GS: Google Scholar
JSUR: Journal of Serendipitous and Unexpected Results
OHBM: Organization for Human Brain Mapping
PoP: Publish or Perish software
WoS: Web of Science
