# Supplementary material for: Types of Errors Hiding in Google Scholar Data
Source: J Med Internet Res. 2022 May 27;24(5):e28354. doi: 10.2196/28354 (PMC9187964; doi:10.2196/28354)
Supplement: Multimedia Appendix 2 [file jmir_v24i5e28354_app2.pdf]

# Multimedia Appendix 2

Number of errors detected per reference retrieved from Google Scholar

| N° of errors, n, % | References | Errors |
|---|---|---|
| 0 | 2 (0.7) | 0 (0.0) |
| 1 | 62 (22.1) | 60 (7.9) |
| 2 | 82 (29.2) | 152 (20.1) |
| 3 | 89 (31.7) | 261 (34.6) |
| 4 | 25 (8.9) | 84 (11.1) |
| 5 | 12 (4.3) | 85 (11.3) |
| 6 | 7 (2.5) | 78 (10.3) |
| 7 | 2 (0.7) | 35 (4.6) |
| Total | 281 (100.0) | 755 (100.0) |
