# Supplementary material for: Types of Errors Hiding in Google Scholar Data
Source: J Med Internet Res. 2022 May 27;24(5):e28354. doi: 10.2196/28354 (PMC9187964; doi:10.2196/28354)
Supplement: Multimedia Appendix 3 [file jmir_v24i5e28354_app3.pdf]

# Multimedia Appendix 3

Data collection error: types and rates

| Type of errors, n, % | N° errors | Error rate (%)/N° references | Error rate (%)/Total N° of errors |
|---|---|---|---|
| Duplicate | 16 (38.1) | 5.7 | 2.1 |
| Translation/Reprint | 6 (14.3) | 2.1 | 0.8 |
| Unvailable/Inaccessible | 2 (4.8) | 0.7 | 0.3 |
| URL missing | 18 (42.9) | 6.4 | 2.4 |
| Total | 42 (100.0) | 11.7 | 5.6 |
