# Supplementary material for: Types of Errors Hiding in Google Scholar Data
Source: J Med Internet Res. 2022 May 27;24(5):e28354. doi: 10.2196/28354 (PMC9187964; doi:10.2196/28354)
Supplement: Multimedia Appendix 4 [file jmir_v24i5e28354_app4.pdf]

Multimedia Appendix 4

Document types of references collected from Google Scholar as a function of 3 categories (academic journal, nonacademic journal, and Google Scholar non-"scholarly literature").

Type of reference, n, %	Academic publications	Nonacademic publications	GS Non-"Scholarly literature"	Total references
Article	114 (40.7)	1 (0.4)		115 (41.1)
Bachelor's or master's thesis		8 (2.9)		8 (2.9)
Blog		3 (1.1)	3 (1.1)	3 (1.1)
Book	53 (18.9)			53 (18.9)
Book chapter	31 (11.1)			31 (11.1)
Book review		4 (1.4)	4 (1.4)	4 (1.4)
Conference proceeding	5 (1.8)			5 (1.8)
Communication poster		1 (0.4)		1 (0.4)
Courses & bibliography		4 (1.4)	4 (1.4)	4 (1.4)
Doctoral thesis		37 (13.2)		37 (13.2)
Editorial		3 (1.1)	3 (1.1)	3 (1.1)
Magazine		5 (1.8)	5 (1.8)	5 (1.8)
Research or technical report		2 (0.7)		2 (0.7)
Talk		1 (0.4)		1 (0.4)
Unpublished manuscript (preprint included)		8 (2.9)		8 (2.9)
Total	203 (72.5)	77 (27.5)	19 (6.8)	280 (100.0)
