# Supplementary material for: Types of Errors Hiding in Google Scholar Data
Source: J Med Internet Res. 2022 May 27;24(5):e28354. doi: 10.2196/28354 (PMC9187964; doi:10.2196/28354)
Supplement: Multimedia Appendix 5 [file jmir_v24i5e28354_app5.pdf]

Multimedia Appendix 5

Results of the Mann-Whitney test on the type of reference (academic publication or nonacademic document) and the number of Google Scholar errors, and data visualization with a box plot.

Mann-Whitney test

Ranks

	Type of reference	N	Mean rank	Sum of ranks
Error type	Academic publication	203	127,21	25823,50
	Nonacademic document	77	175,54	13516,50
	Total	280		

Significance test[a]

	Nombre_erreurs
U of Mann-Whitney	5117,500
W of Wilcoxon	25823,500
Z	-4,599
Asymptotic significance (bilateral)	,000

a. Grouping variable: Type of reference

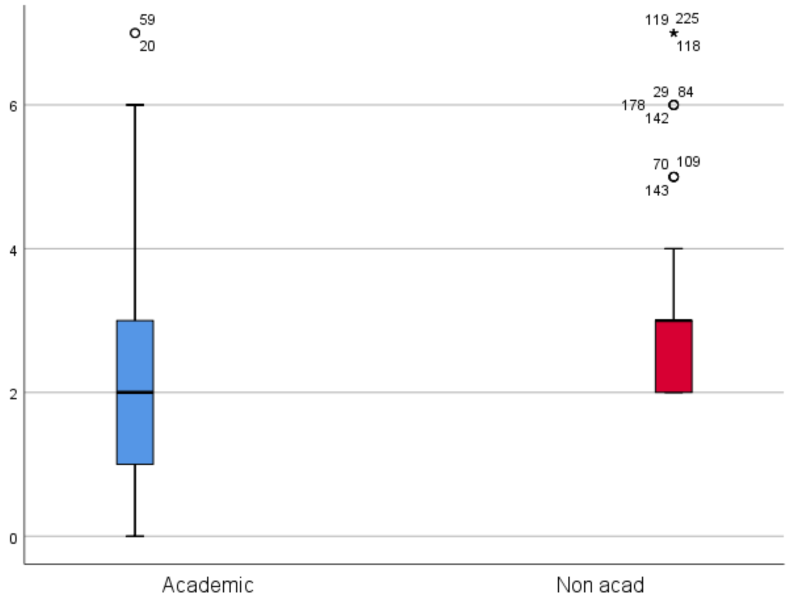
59
20
119 225
118
29 84
178
142
70 109
143
6
4
2
0
Academic
Non acad
