# Supplementary material for: Types of Errors Hiding in Google Scholar Data
Source: J Med Internet Res. 2022 May 27;24(5):e28354. doi: 10.2196/28354 (PMC9187964; doi:10.2196/28354)
Supplement: Multimedia Appendix 6 [file jmir_v24i5e28354_app6.pdf]

Multimedia Appendix 6

Inaccurate content identified in the “Author” column retrieved from Google Scholar via Publish or Perish software.

Type of errors, n, %	N° errors	Error rate (%)/N° references	Error rate (%)/Total N° of errors
Missing authors	41 (67.2)	15.0	5.4
Added authors	10 (16.4)	3.7	1.3
Missing part of the author’s name	1 (1.6)	0.4	0.1
Initials errors	5 (8.2)	1.8	0.7
Replacement of authors by a book or a book chapter title	3 (4.9)	1.1	0.4
Replacement of authors by a journal name	1 (1.6)	0.4	0.1
Total	61 (100.0)	19.4	8.1
