# Supplementary material for: Types of Errors Hiding in Google Scholar Data
Source: J Med Internet Res. 2022 May 27;24(5):e28354. doi: 10.2196/28354 (PMC9187964; doi:10.2196/28354)
Supplement: Multimedia Appendix 7 [file jmir_v24i5e28354_app7.pdf]

Multimedia Appendix 7

Inaccurate content identified in the "Title" column retrieved from Google Scholar via Publish or Perish software.

Type of errors, n, %	N° errors	Error rate (%)/N° references	Error rate (%)/Total N° of errors
Spelling error	1 (1.7)	0.4	0.1
Typographical error	3 (5.0)	1.1	0.4
Incorrect title	5 (8.3)	1.8	0.7
Incomplete title	30 (50.0)	10.9	4.0
Replacement of the title by an editor	2 (3.3)	0.7	0.3
Replacement of the book title by one of the book chapter titles	11 (18.3)	4.0	1.5
Replacement of the thesis title by one of the thesis chapter titles	2 (3.3)	0.7	0.3
Replacement of the title by the domain name of the website hosting the document	2 (3.3)	0.7	0.3
Irrelevant part added	4 (6.7)	1.5	0.5
Total	60 (100.0)	20.8	7.9
