# Supplementary material for: Types of Errors Hiding in Google Scholar Data
Source: J Med Internet Res. 2022 May 27;24(5):e28354. doi: 10.2196/28354 (PMC9187964; doi:10.2196/28354)
Supplement: Multimedia Appendix 8 [file jmir_v24i5e28354_app8.pdf]

Multimedia Appendix 8

Inaccurate content identified in the “Year” column retrieved from Google Scholar via Publish or Perish.

Type of errors, n, %	N° errors	Error rate (%)/N° references	Error rate (%)/Total N° of errors
"0"	22 (71.0)	8.0	2.9
+ 1 an	2 (6.5)	0.7	0.3
- 1 an	4 (12.9)	1.5	0.5
- 3 ans	1 (3.2)	0.4	0.1
- 7 ans	1 (3.2)	0.4	0.1
- 100 ans	1 (3.2)	0.4	0.1
Total	31 (100.0)	11.3	4.1
