# Supplementary material for: Types of Errors Hiding in Google Scholar Data
Source: J Med Internet Res. 2022 May 27;24(5):e28354. doi: 10.2196/28354 (PMC9187964; doi:10.2196/28354)

Multimedia Appendix 9

Content of the "Publication" column retrieved from Google Scholar via Publish or Perish as a function of reference document type.

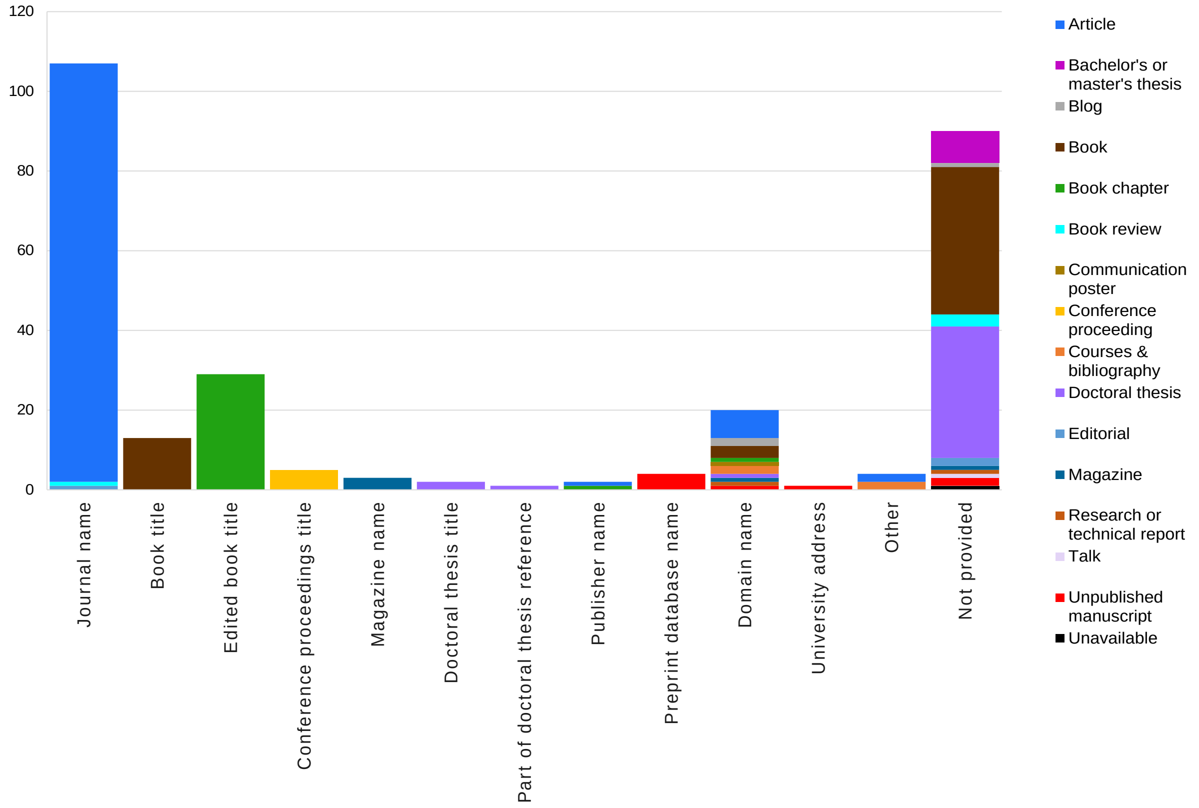

Supplement: Multimedia Appendix 9 [file jmir_v24i5e28354_app9.pdf]
