# Supplementary material for: Types of Errors Hiding in Google Scholar Data
Source: J Med Internet Res. 2022 May 27;24(5):e28354. doi: 10.2196/28354 (PMC9187964; doi:10.2196/28354)
Supplement: Multimedia Appendix 10 [file jmir_v24i5e28354_app10.pdf]

Multimedia Appendix 10

Spelling and orthographical errors in the academic publications retrieved from Google Scholar via Publish or Perish software: journal name, edited book title, and conference proceedings book title.

Type of errors, n, %	N° errors	Error rate (%)/N° references
**Journal name error**	**68 (100.0)**	**21.1**
Incorrect abbreviation	1 (1.5)	0.4
Capitalization error	34 (50.0)	12.1
Irrelevant part added	1 (1.5)	0.4
Incomplete name	31 (45.6)	11.1
Incorrect name	1 (1.5)	0.4
**Title error**	**33 (100.0)**	**11.8**
Incomplete edited book title	26 (78.8)	9.3
Incomplete conference proceeding book title or incomplete edited book title	5 (15.2)	1.8
Incorrect edited book title	2 (6.1)	0.7
